# Artificial neural network-based models used for predicting 28- and 90-day mortality of patients with hepatitis B-associated acute-on-chronic liver failure

**DOI:** 10.1186/s12876-020-01191-5

**Published:** 2020-03-13

**Authors:** Yixin Hou, Qianqian Zhang, Fangyuan Gao, Dewen Mao, Jun Li, Zuojiong Gong, Xinla Luo, Guoliang Chen, Yong Li, Zhiyun Yang, Kewei Sun, Xianbo Wang

**Affiliations:** 1 Center of Integrative Medicine, Beijing Ditan Hospital, Capital Medical University, Beijing, 100015 People’s Republic of China; 2 Department of Hepatology, The First Hospital Affiliated to Hunan University of Chinese Medicine, Changsha, Hunan 410007 People’s Republic of China; 3 Department of Hepatology, The First Affiliated Hospital of Guangxi University of Chinese Medicine, Nanning, Guangxi 530021 People’s Republic of China; 4 Center of Integrative Medicine, Beijing 302 Hospital, Beijing, 100039 People’s Republic of China; 5 Department of Infectious Diseases, Renmin Hospital of Wuhan University, Wuhan, Hubei 430060 People’s Republic of China; 6 Department of Hepatology, Hubei Provincial Hospital of Traditional Chinese Medicine, Wuhuan, Hubei 430061 People’s Republic of China; 7 Department of Hepatology, Xiamen Hospital of Traditional Chinese Medicine, Xiamen, Fujian 361009 People’s Republic of China; 8 Department of Hepatology, The Affiliated Hospital of Shandong University of Traditional Chinese Medicine, Jinan, Shandong 250014 People’s Republic of China

**Keywords:** Hepatitis B virus, Acute-on-chronic liver failure, Short-term, Mortality, Prognosis, Artificial neural network

## Abstract

**Background:**

This study aimed to develop prognostic models for predicting 28- and 90-day mortality rates of hepatitis B virus (HBV)-associated acute-on-chronic liver failure (HBV-ACLF) through artificial neural network (ANN) systems.

**Methods:**

Six hundred and eight-four cases of consecutive HBV-ACLF patients were retrospectively reviewed. Four hundred and twenty-three cases were used for training and constructing ANN models, and the remaining 261 cases were for validating the established models. Predictors associated with mortality were determined by univariate analysis and were then included in ANN models for predicting prognosis of mortality. The receiver operating characteristic curve analysis was used to evaluate the predictive performance of the ANN models in comparison with various current prognostic models.

**Results:**

Variables with statistically significant difference or important clinical characteristics were input in the ANN training process, and eight independent risk factors, including age, hepatic encephalopathy, serum sodium, prothrombin activity, γ-glutamyltransferase, hepatitis B e antigen, alkaline phosphatase and total bilirubin, were eventually used to establish ANN models. For 28-day mortality in the training cohort, the model’s predictive accuracy (AUR 0.948, 95% CI 0.925–0.970) was significantly higher than that of the Model for End-stage Liver Disease (MELD), MELD-sodium (MELD-Na), Chronic Liver Failure-ACLF (CLIF-ACLF), and Child-Turcotte-Pugh (CTP) (all *p* < 0.001). In the validation cohorts the predictive accuracy of ANN model (AUR 0.748, 95% CI: 0.673–0.822) was significantly higher than that of MELD (*p* = 0.0099) and insignificantly higher than that of MELD-Na, CTP and CLIF-ACLF (*p* > 0.05). For 90-day mortality in the training cohort, the model’s predictive accuracy (AUR 0.913, 95% CI 0.887–0.938) was significantly higher than that of MELD, MELD-Na, CTP and CLIF-ACLF (all *p* < 0.001). In the validation cohorts, the prediction accuracy of the ANN model (AUR 0.754, 95% CI: 0.697–0.812 was significantly higher than that of MELD (*p* = 0.019) and insignificantly higher than MELD-Na, CTP and CLIF-ACLF (*p* > 0.05).

**Conclusions:**

The established ANN models can more accurately predict short-term mortality risk in patients with HBV- ACLF.

The main content has been postered as an abstract at the AASLD Hepatology Conference (10.1002/hep.30257).

## Background

Hepatitis B virus (HBV) infects approximate 240 million people worldwide and particularly 93 million in China [1, 2]. HBV has become one of the leading causes for acute-on-chronic liver failure (ACLF), mainly characterized as a rapid deterioration of liver function with a high short-term mortality [3]. In China, HBV-associated ACLF (HBV-ACLF) accounts for more than 80% of the whole ACLF cases and would cause a high mortality rate (60–80%) if left without effective treatment (e.g. transplantation) [4]. Liver transplantation is currently the most effective therapeutic option for HBV-ACLF. However, due to shortage of liver donors and some socioeconomic problems, liver transplantation is limited in clinics [5]. To decrease mortality of HBV-ACLF, it is vital to accurately identify patients with poor prognosis so as to take treatment as early, including prior organ allocation from limited liver donors.

At present, there have actually no ideal models to predict short-term outcomes of patients with HBV-ACLF yet. Model of end-stage liver disease (MELD) and the modified type--MELD integrating sodium (MELD-Na)--have been gradually proposed [6–8] to predict prognosis of patients with ACLF, with moderate accuracy [9]. However, due to limited predictive accuracy, these scoring systems are still unsatisfactory [9, 10]. More accurate prognostic models are urgent.

It is important to combine more kinds of important parameters to construct models to predict short-term mortality of ACLF. The artificial neural networks (ANNs), structurally and functionally mimic biological neural systems, have been widely using to manage nonlinear complex biological systems. For example, ANN models have been used to predict post-hepatectomy survival of early hepatocellular carcinoma [11], in-hospital mortality of type 2 diabetes after major lower extremity amputation [12], neuroblastoma patients’ outcome [13], and cardiac complications following posterior lumbar spine fusion [14]. It has been revealed that the ANN models are more accurate than multiple logistic regression and multiple linear discriminant analysis models [15, 16]. In this study, we established ANN-based models to predict 28- and 90- day mortality of HBV-ACLF.

## Methods

### Patient characteristics

From January 2008 to May 2016, a total of 2532 cases of patients with chronic HBV (CHB)-ACLF who were hospitalized for an acute deterioration of liver function at the Beijing Ditan Hospital, Capital Medical University (Beijing, China), First Hospital Affiliated to Hunan University of Chinese Medicine (Changsha, China), First Affiliated Hospital of Guangxi University of Chinese Medicine (Nanning, China), Affiliated Hospital of Shandong University of Traditional Chinese Medicine (Jinan, China), Beijing 302 Hospital (Beijing, China), Renmin Hospital Hospital of Wuhan University (Wuhan, China), Hubei Provincial Hospital of Traditional Chinese Medicine (Wuhan, China), and Xiamen Hospital of Traditional Chinese Medicine (Xiamen, China) were retrospective reviewed. All the above hospital were tertiary hospitals in China. Patients’ inclusion criteria were: 1) patients were diagnosed with HBV-ACLF, 2) patients received standard medical management (except liver transplantation), including absolute bed rest, energy and vitamin supplement, maintenance water, intravenous infusions of albumin, electrolyte and acid-base correction, and prevention and management of complications, 3) patients received antiviral therapy (i.e. administration of lamivudine, adefovir dipivoxil, entecavir, telbivudine and tenofovir) based on the HBV replication levels, and the financial condition and willingness of patients, and 4) patients were followed up from their diagnosis until either the end of the 28- or 90-day follow-up period or their death in hospital. Patients who had: 1) hepatocellular carcinoma or other liver malignancy, 2) other organ malignancies that might influence treatment outcomes, 3) infection of hepatitis A, C, D, or E virus, 4) other virus infection (such as cytomegalovirus and human immunodeficiency virus co-infection), 5) autoimmune liver disease, 6) liver decompensated cirrhosis, or 7) severe chronic extrahepatic diseases (such as cardiovascular diseases, diabetes, kidney diseases), were excluded. Patients who were pregnancy or failed to meet the Asia Pacific Association for the Study of the Liver (APASL) criteria for ACLF were also excluded. Eventually, 684 cases of patients who were diagnosed with HBV-ACLF at the admission day or within 90 days after admission were analyzed; 423 of them from the Beijing Ditan Hospital were used for training and constructing ANN-based models and the remaining 261 from other hospitals were for validating the established models. All the involved centers were tertiary hospitals, with reliable treatment conditions and levels. In addition, if there was any difference in the units, presentation meas, etc. of parameters among different hospitals, they would be uniformly transformed. All these could make data measured in different hospitals comparable.

To validate the constructed models, 261 cases, including 103 from the First Hospital Affiliated to Hunan University of Chinese Medicine, 42 from the First Affiliated Hospital of Guangxi University of Chinese Medicine, 14 from the Affiliated Hospital of Shandong University of Traditional Chinese Medicine, 30 from the Beijing 302 Hospital, 32 from the Renmin Hospital of Wuhan University, 25 from the Hubei Provincial Hospital of Traditional Chinese Medicine, and 15 from Xiamen Hospital of Traditional Chinese Medicine, were retrospectively reviewed. The study protocol was in accordance with the ethical guidelines of the Declaration of Helsinki, and was approved by the ethics committees of the above hospitals.

### Diagnosis

CHB was determined as the detection of hepatitis B surface antigen for over 6 months [17]. Cirrhosis was diagnosed on the basis of previous liver biopsy examinations or the following combined parameters [18, 19], including: 1) ultrasonography, computed tomography, and magnetic resonance imaging characteristics of a small liver with or without splenomegaly/ascites; 2) an aspartate aminotransferase (AST)-platelet ratio index score > 2; and 3) an albumin level < 35 g·L^− 1^, without other definite reasons of hypoalbuminemia, such as renal and gastrointestinal loss. Organ failure was defined according to the CLIF-COF score [20], including liver failure with total bilirubin (TBIL) ≥ 12 mg·dL^− 1^, renal failure with creatinine ≥2 mg·dL^− 1^ or a requirement of renal support therapy, cerebral failure with hepatic encephalopathy (HE) grade III/IV, coagulation failure with an international normalized ratio (INR) ≥ 2.5, circulatory failure with a requirement of vasoconstrictors for maintenance of arterial pressure, and respiratory failure with SpO_2_/FiO_2_ ≤ 214 or PaO_2_/FiO_2_ ≤ 200.

ACLF was defined on the basis the APASL criteria as follows (3): 1) acute hepatic damage manifesting as jaundice, TBIL ≥5 mg·dL^− 1^ (85 μM) and coagulopathy, 2) with INR ≥ 1.5 or prothrombin activity (PTA) < 40%, 3) complicated with clinical ascites and/or HE within 4 weeks in patients who were previously diagnosed or undiagnosed chronic liver disease. Chronic Liver Failure-ACLF (CLIF-ACLF), MELD, MELD-Na, and Child-Turcotte-Pugh (CTP) scores were calculated on the basis of previously published criteria, respectively. All scores and definitions were applied upon the enrollment in this study.

### Data collection

Patients’ demographics, laboratory parameters, clinical variables (HBV reactivation, bacterial infection, gastrointestinal hemorrhage, hepatotoxic drugs, active alcoholism, and surgery), complications (HE, hepatorenal syndrome, hyponatremia, and spontaneous bacterial peritonitis), and organ (liver, renal, brain, coagulation, circulatory, and respiratory) failure were collected from patients’ medical records and the hospitals’ databases upon the HBV-ACLF diagnosis and during hospitalization. Four scoring systems related to clinical prognosis, including MELD, MELD-Na, CLIF-ACLF and CTP scores, were assessed at baseline. Patients receiving liver transplantation were taken as lost to follow-up, and mortality rate was estimated as transplant-free rate. Mortality at 28 and 90 days after enrollment was confirmed from patients’ medical records or through direct contact with their families, and mortality rates were thus calculated accordingly.

### Construction of ANN

ANN comprises highly complicated and interconnected processing units (neurones) related to weighted connections, with an input, an output, and one or more hidden layers [21–24]. The advantages of ANN include self-learning, self-adapting and inference processes. By learning from examples, ANN connect each input with the corresponding output through changing the weight of the connections within neurones. When applied, an input will be propagated from the first layer of neurones via each upper layer till an output is generated, followed by a self-adapting process. The value of the produced output is compared with that of the desired one. If there is a discrepancy between these two values, an error signal will then be produced and accordingly, a back propagation (BP) method should be used to modify the weight of the interneurone connections to reduce the total error of the network. During the learning process, the error between the values of the produced and desired outputs is decreasing until a minimum is reached (i.e. convergence of the network). Thereafter, inference process is performed, where the outputs (prognosis) can be produced from new input data on the basis of the knowledge accumulated during training process. Therefore, the ANN can accurately carry out predictions on data sets [21–24].

In this study, 28- and 90-day mortality in the 423 HBV-ACLF input layers contained neurones that imported the data available, including various clinical, demographic and laboratory data, and the output layers comprised neurones that exported the corresponding predictive outcomes. The hidden layers were applied to make complicated interactions between the input and output neurones. Variables significantly associated with prognosis of HBV-ACLF patients were included to construct ANNs by using Mathematica 11.1.1 for Microsoft windows (64-bit) (April 18, 2017), a graphical neural network development tool. Six hundred and eight-four eligible patients were allocated to a training cohort (*n* = 423, 61.8%) or validation cohort (*n* = 261, 38.2%).

The learning process of this ANN was carried out by BP through assessing the errors between the values of the generated and desired outputs. The interneurone connections was adjusted the weight to reduce the overall errors of the network. Learning (training) would be stopped if the total of square errors reached minimum in comparison with the cross-validation data set. Eventually, the final form provided the 28- and 90-day mortality risks in each patient.

### Statistical analysis

All statistical analysis were conducted by using SPSS software (Version 19.0, IBM, Armonk, NY, USA). The Kolmogorov-Smirnov test was used to evaluate whether the sample data was normally distributed. Patients were assigned to survivor and non-survivor groups, respectively at 28 and 90 days during the follow-up. For continuous variables, data were presented as mean ± standard deviation or median with interquartile range, and were analyzed with the Student’s *t*-test or Mann-Whitney U test between survivor and non-survivor groups. For categorical variables, data were presented as frequencies or percentages, and were then compared between groups as appropriate using the Pearson chi-square or Fisher’s exact test. After the association of the demographic, biochemical and clinical variables (inputs) with prognosis (outputs) was determined, the variables with statistically significant difference or important clinical characteristics were selected as the input layers to construct ANNs for prediction of 28- and 90-day mortality of patients with HBV-ACLF. Odds ratio (OR) and the corresponding 95% confidence interval (95% CI) were determined.

Prediction performances of the ANN models both in the training and validation cohorts were assessed by receiver operating characteristic curve (ROC) analysis, respectively, where area under the ROC curve (AUR) was applied to compare the ANN’s prediction performance with that of MELD, MELD-Na, CTP and CLIF-ACLF, respectively using Mann-Whitney U test. *p* < 0.05 was considered statistical difference.

## Results

### Baseline characteristics of patients

Six hundred and eight-four cases of patients diagnosed with HBV-ACLF were eventually reviewed in the study, with a mean age of 43.9 ± 11.7 years and a male proportion of 85.1%. The basic characteristics, biochemical parameters and some scoring systems (such as MELD) of patients were shown in Table 1. One hundred and seventy-five patients (25.6%) died during a 28-day follow-up and 251 patients (36.7%) died during a 90-day follow-up. The mean MELD, MELD-Na, CTP and CLIF-ACLF scores of the total study population were 22.9 (20.0, 26.5), 22.3 (18.1, 28.0), 11 (10, 12), and 38.6 ± 8.1, respectively.

**Table 1:** Basic clinical characteristics of patients with HBV- ACLF

Patient’s Characteristics	All patients (*n* = 684)	Training cohort (*n* = 423)	Validation cohorts (*n* = 261)	*P* value
Age (yr)	43.9 ± 11.6	43.8 ± 11.4	43.9 ± 11.6	0.440
Male %	582 (85.1)	349 (82.5)	233 (89.3)	0.016
Complications				
Hyponatremia (n/%)	242 (35.4)	175 (41.4)	67 (25.7)	< 0.001
Astices (n/%)	405 (59.2)	279 (66.0)	126 (48.3)	0.010
Spontaneous bacterial peritonitis (n/%)	82 (12.0)	32 (7.5)	50 (19.2)	< 0.001
HE (n/%)	122 (17.8)	98 (23.2)	24 (9.2)	
HE grade I-II (n/%)	92 (13.5)	76 (18.0)	16 6.1)	< 0.001
HE grade III-IV (n/%)	25 (3.7)	22 (5.2)	3 (1.1)	0.006
Hepatorenal syndrome (n/%)	41 (6.0)	38 (8.9)	3 (1.1)	< 0.001
Organ failures				
Liver (n/%)	559 (87.6)	326 (77.1)	233 (89.3)	< 0.001
Kidney (n/%)	23 (3.4)	19 (4.5)	4 (1.5)	0.037
Brain (n/%)	26 (3.8)	22 (5.2)	4 (1.5)	0.015
Coagulation (n/%)	158 (23.1)	100 (23.6)	58 (22.2)	0.669
Circulatory (n/%)	2 (0.2)	2 (0.5)	0 (0)	0.266
Respiratory (n/%)	1 (0.1)	1 (0.2)	0 (0)	0.432
Treatment with NUCs	597 (87.3%)	377 (89.1%)	220 (84.3%)	0.065
Lamivudine alone (n/%)	245 (35.8%)	112 (26.5%)	133 (60.0%)	< 0.001
Entecavir alone (n/%)	217 (31.7%)	209 (49.4%)	8 (3.1%)	< 0.001
Adefovir alone (n/%)	79 (11.5%)	17 (4.0%)	62 (23.8%)	< 0.001
Telbivudine alone (n/%)	36 (5.2%)	3 (0.7%)	3 (1.1%)	0.549
Tenofovir alone (n/%)	0 (0.%)	0 (0%)	0 (0%)	–
≥ 2 types of NUCs (n/%)	50 (7.3%)	36 (8.5%)	14 (5.4%)	0.125
Laboratory data				
ALT (U·L^−1^)	347.9 (137.1, 829)	443.2 (193.7, 939.0)	245.0 (91.0, 582.8)	< 0.001
AST (U·L^−1^)	298.1 (139.5, 611.9)	360.1 (176.3, 746.1)	197.0 (102.0, 401.0)	< 0.001
TBIL (μmo·L^−1^)	323.5 ± 148.4	318.3 ± 149.3	331.9 ± 146.7	0.196
Albumin (g·L^− 1^)	31.0 ± 4.9	31.2 ± 4.8	30.6 ± 4.9	0.982
GGT (U·L^− 1^)	84 (52.0, 136.0)	83.9 (52.3, 135.3)	85.0 (51.0, 136.0)	0.905
ALP (U·L^−1^)	138.5 (109.9172.8)	129.6 (105.1, 165.8)	156.4 ± 48.2	< 0.001
Cholinesterase (U·L^−1^)	3080.7 ± 1659.9	3313.5 ± 1468.1	2510.0 (1426.0,3687)	< 0.001
PTA (%)	30.1 ± 9.6	28.6 ± 10.2	32.6 ± 8.0	< 0.001
INR	2.3 ± 0.8	2.0 (1.8,2.5)	2.1 (1.8,2.5)	0.107
White blood cell (× 10^9^·L^−1^)	6.6 (4.9, 8.9)	7.0 (5.2, 9.5)	6.4 (4.6, 8.1)	0.044
Neutrophil count (× 10^9^·L^−1^)	4.5 (3.2, 6.9)	4.7 (3.4, 7.4)	3.8 (2.6, 5.7)	0.193
Lymphocyte count (×10^9^·L^−1^)	1.3 (0.9, 1.8)	1.3 (0.9, 1.8)	–	–
NLR	3.7 (2.4, 6.1)	3.7 (2.4, 6.1)	–	–
Platelet count (×10^9^·L^−1^)	107.8 ± 52.4	114.4 ± 53.5	97.1 ± 48.6	0.500
Serum potassium (mmol·L^− 1^)	4.0 ± 0.6	4.0 ± 0.6	4.0 ± 0.5	0.051
Serum sodium (mmol·L^− 1^)	135.4 ± 4.8	135.4 ± 4.7	136.0 (133.0,138.0)	0.961
Serum creatinine (mg·dL^− 1^)	69.2 (58.0, 83.9)	66.8 (57.4, 82.1)	72.0 (61.0, 84.8)	0.061
HBeAg positive rate (n/%)	370 (54.1)	265 (62.6)	105 (40.2)	< 0.001
HBV-DNA level (Log_10_ copies·mL^−1^)	5.8 ± 1.5	5.8 ± 1.5	5.7 ± 1.7	0.601
Mortality				
28- day (n/%)	175 (25.6)	132 (31.2)	43 (16.5)	< 0.001
90- day (n/%)	251 (36.7)	173 (40.9)	78 (29.9)	0.004
Scoring systems				
CTP	11 (10, 12)	11 (10, 12)	10 (9, 11)	< 0.001
CLIF-ACLF	38.6 ± 8.1	39.3 ± 18.8	37.6 ± 6.7	< 0.001
MELD	22.9 (20.0, 26.5)	22.6 (19.5, 26.5)	23.3 (20.8, 27.0)	0.065
MELD-Na	22.3 (18.1, 28.0)	23.0 (18.9, 28.6)	21.1 (17.0, 27.6)	0.005

Data were presented as n (%), mean ± standard deviation, or median (interquartile range) as appropriate. *ACLF* acute-on chronic liver failure, *HBV* hepatitis B virus, *NUCs* nucleotide analogs, *HE* hepatic encephalopathy, *ALT* alanine aminotransferase, *AST* aspartate aminotransferase, *TBIL* total bilirubin, *GGT* gamma-glutamyl transpeptidase, *ALP* alkaline phosphatase, *PTA* prothrombin activity, *INR* international normalized ratio, *NLR* neutrophil to lymphocyte ratio, *HBeAg* hepatitis B e antigen, *CTP* Child-Turcotte-Pugh, *CLIF-ACLF* Chronic Liver Failure-ACLF, *MELD* model of end-stage liver disease, *MELD-Na* MELD integrating sodium

### Construction of ANN models

Patients were assigned to survivor and non-survivor groups, respectively at 28 and 90 days during the follow-up. PTA (OR = 0.908, 95% CI: 0.891–0.926, *p* < 0.001), TBIL (OR = 1.003, 95% CI: 1.002–1.004, *p* < 0.001), age (OR = 1.037, 95% CI: 1.022–1.053, *p* < 0.001), serum sodium (OR = 0.923, 95% CI: 0.892–0.955, *p* < 0.001), alkaline phosphatase (ALP) (OR = 0.995, 95% CI: 0.991–0.999, *p* = 0.009), gamma-glutamyl transpeptidase (GGT) (OR = 0.996, 95% CI: 0.993–0.999, *p* = 0.006), HE (OR = 5.623, 95% CI: 2.358–10.891, *p* < 0.001) and hepatitis B e antigen (HBeAg) positive rate (OR = 0.616, 95% CI: 0.429–0.884, *p* = 0.009) were significantly associated with 28-day mortality (Table 2). All these variables were included to build an ANN model.

**Table 2:** Univariate analysis of 28- and 90-day mortality of patients with HBV- ACLF

Patient’s Characteristics	Univariate analysis (28-day mortality)			Univariate analysis (90-day mortality)		
	β	OR (95% CI)	*p* value	β	OR (95% CI)	*P* value
Age (yr)	0.037	1.037 (1.022–1.053)	< 0.001	0.039	1.040 (1.026–1.054)	< 0.001
Male %	−0.056	0.946 (0.608–1.472)	0.805	−0.048	0.953 (0.646–1.406)	0.810
Complications						
Hyponatremia (%)	0.573	1.773 (1.259–2.496)	0.001	0.687	1.977 (1.465–2.668)	< 0.001
Astices (%)	0.515	1.673 (1.132–2.472)	0.010	0.560	1.751 (1.243–2.465)	0.001
Spontaneous bacterial peritonitis (%)	0.879	2.409 (1.406–4.012)	0.001	0.733	2.080 (1.291–3.353)	0.003
HE	1.181	5.623 (2.358–10.891)	< 0.001	1.220	2.235 (1.883–4.837)	< 0.001
Hepatorenal syndrome (%)	1.214	3.368 (2.162–5.248)	< 0.001	1.277	3.585 (2.413–5.326)	< 0.001
Organ failures						
Liver (%)	1.100	3.005 (1.726–5.230)	< 0.001	1.104	3.016 (1.872–4.860)	< 0.001
Kidney (%)	1.248	3.483 (1.921–6.316)	< 0.001	1.492	4.446 (2.685–7.363)	< 0.001
Brain (%)	1.987	7.295 (4.400–12.094)	< 0.001	1.955	7.067 (4.394–11.364)	< 0.001
Coagulation (%)	1.377	3.963 (2.808–5.593)	< 0.001	1.197	3.309 (2.453–4.499)	< 0.001
Circulatory (%)	3.034	20.775 (4.975–86.754)	< 0.001	3.034	20.775 (4.975–86.754)	< 0.001
Respiratory (%)	2.457	11.673 (1.599–85.199)	0.015	2.457	11.673 (1.599–85.199)	0.015
Treatment with NUCs (%)	−0.547	0.388 (0.249–0.604)	< 0.001	−0.870	0.419 (0.281–0.625)	< 0.001
Lamivudine alone (%)	−0.234	0.791 (0.527–1.189)	0.259	−0.127	0.881 (0.624–1.243)	0.470
Entecavir alone (%)	−0.079	0.924 (0.657–1.300)	0.649	−0.164	0.848 (0.629–1.144)	0.281
Adefovir alone (%)	−0.159	0.853 (0.349–2.085)	0.727	−0.439	0.644 (0.265–1.569)	0.333
Telbivudine alone (%)	−3.013	0.049 (0.000–208.362)	0.480	−0.361	0.697 (0.098–4.975)	0719
≥ 2 NUCs (%)	−0.329	0.719 (0.366–1.416)	0.340	−0.079	0.924 (0.544–1.569)	0.770
Laboratory data						
ALT (U·L^−1^)	0.000	1.000 (1.000–1.000)	0.520	0.000	1.000 (1.000–1.000)	0.478
AST (U·L^−1^)	0.000	1.000 (1.000–1.000)	0.533	0.000	1.000 (1.000–1.000)	0.900
TBIL (μmol·L^−1^)	0.003	1.003 (1.002–1.004)	< 0.001	0.003	1.003 (1.003–1.004)	< 0.001
Albumin (g·L^−1^)	−0.028	0.972 (0.938–1.008)	0.124	−0.056	0.946 (0.917–0.976)	0.001
GGT (U·L^−1^)	−0.004	0.996 (0.993–0.999)	0.006	−0.004	0.997 (0.994–0.999)	0.005
ALP (U·L^−1^)	−0.005	0.995 (0.991–0.999)	0.009	−0.003	0.997 (0.993–1.000)	0.034
Cholinesterase (U·L^−1^)	0.000	1.000 (1.000–1.000)	0.277	0.000	1.000 (1.000–1.000)	0.008
PTA (%)	−0.096	0.908 (0.891–0.926)	< 0.001	− 0.081	0.922 (0.907–0.937)	< 0.001
INR	0.785	2.193 (1.936–2.485)	< 0.001	0.773	2.166 (1.915–2.448)	< 0.001
White blood cell (×109·L^−1^)	0.082	1.085 (1.050–1.121)	< 0.001	0.083	1.087 (1.057–1.118)	0.083
Neutrophil count (×109·L^−1^)	0.091	1.096 (1.059–1.133)	< 0.001	0.092	1.097 (1.066–1.129)	< 0.001
Lymphocyte count (× 109·L^−1^)	− 0.364	0.695 (0.521–0.928)	0.014	−0.288	0.750 (0.587–0.958)	0.021
NLR	0.128	1.136 (1.078–1.198)	< 0.001	–	–	–
Platelet count (×109·L^−1^)	−0.002	0.998 (0.995–1.002)	0.371	0.148	1.159 (1.094–1.229)	< 0.001
Serum potassium (mmol·L^−1^)	0.361	1.435 (1.140–1.807)	0.002	−0.002	0.998 (0.995–1.001)	0.298
Serum sodium (mmol·L^−1^)	−0.080	0.923 (0.892–0.955)	< 0.001	0.321	1.379 (1.113–1.708)	0.003
Serum creatinine (mg·dL^−1^)	0.005	1.005(1.003–1.006)	< 0.001	−0.096	0.909 (0.882–0.937)	< 0.001
HBeAg positive rate	−0.485	0.616(0.429–0.884)	0.009	0.005	1.005 (1.003–1.006)	< 0.001
HBV-DNA level (Log_10_copies·mL^−1^)	0.031	1.032 (0.918–1.160)	0.602	−0.485	0.616 (0.448–0.847)	0.003

*OR* odds ratio, *95% CI* 95% confidence interval, *HBV* hepatitis B virus, *ACLF* acute-on chronic liver failure, *NUCs* nucleotide analogs, *HE* hepatic encephalopathy, *ALT* alanine aminotransferase, *AST* aspartate aminotransferase, *TBIL* total bilirubin, *GGT* gamma-glutamyl transpeptidase, *ALP* alkaline phosphatase, *PTA* prothrombin activity, *INR* international normalized ratio, *MELD* model of end-stage liver disease, *MELD-Na* MELD integrating sodium, *HBeAg* hepatitis B e antigen

In addition, PTA (OR = 0.922, 95% CI: 0.907–0.937, *p* < 0.001), TBIL (OR = 1.003, 95% CI: 1.003–1.004, *p* < 0.001), age (OR = 1.040, 95% CI: 1.026–1.054, *p* < 0.001), sodium (OR = 1.379, 95% CI: 1.113–1.708, *p* = 0.003), ALP (OR = 0.997, 95% CI: 0.993–1.000, *p* = 0.034), GGT (OR = 0.997, 95% CI: 0.994–0.999, *p* = 0.005), HE (OR = 2.235, 95% CI: 1.883–4.837, *p* < 0.001) and HBeAg positive rate (OR = 1.005, 95% CI: 1.003–1.006, *p* < 0.001) were significantly associated with 90-day mortality. Similarly, these variables were also included to build an ANN model.

**Fig. 1:**
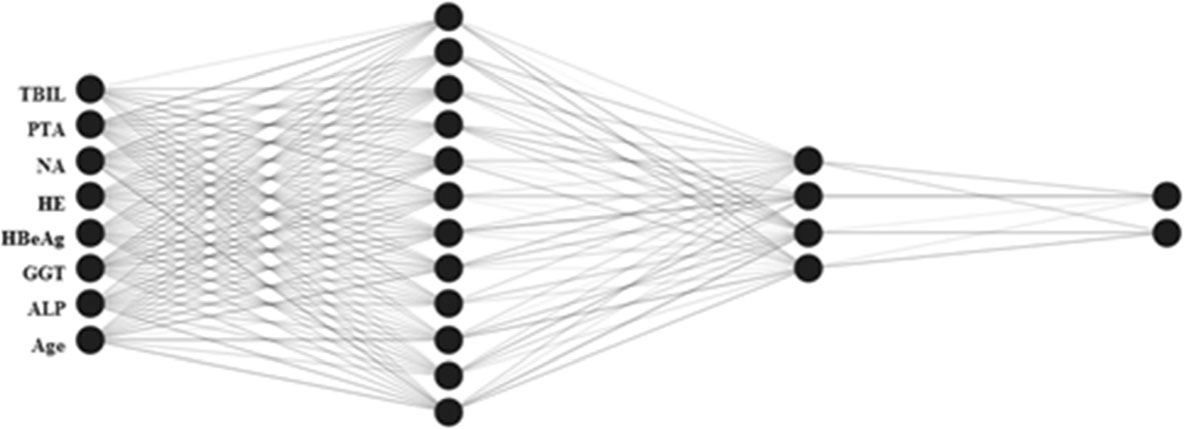
An ANN model for predicting 28-day mortality of patients with HBV-ACLF

**Fig. 2:**
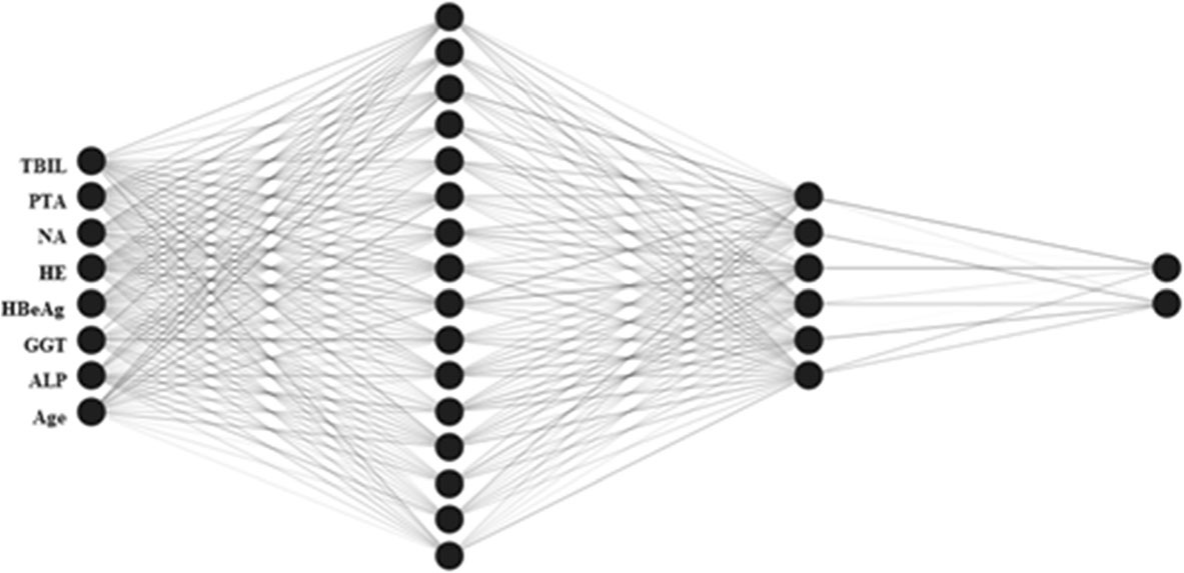
An ANN model for predicting 90-day mortality of patients with HBV-ACLF

As one of the most recognized ANN architectures, multilayer perceptron (MLP) including the input, output and hidden layers were used to establish ANN models. Neurones were shown linkage with weighted connections (Figs. 1 and 2). Generally, the numbers of input and output variables were respectively consistent with those of demographic, biochemical and clinical parameters and those of the set prognosis. As shown in Figs. 1 and 2, the MLP comprises eight input neurones and one output neurone. Following many times of debugging and testing, two hidden neurones were included in the hidden layers in order to increase the performance of MLP. The ANN models for 28- and 90-day mortality of HBV-ACLF patients were shown in Figs. 1 and 2, respectively.

### Evaluation of the predictive accuracy of ANN models for 28- and 90-day mortality of HBV-ACLF

**Fig. 3:**
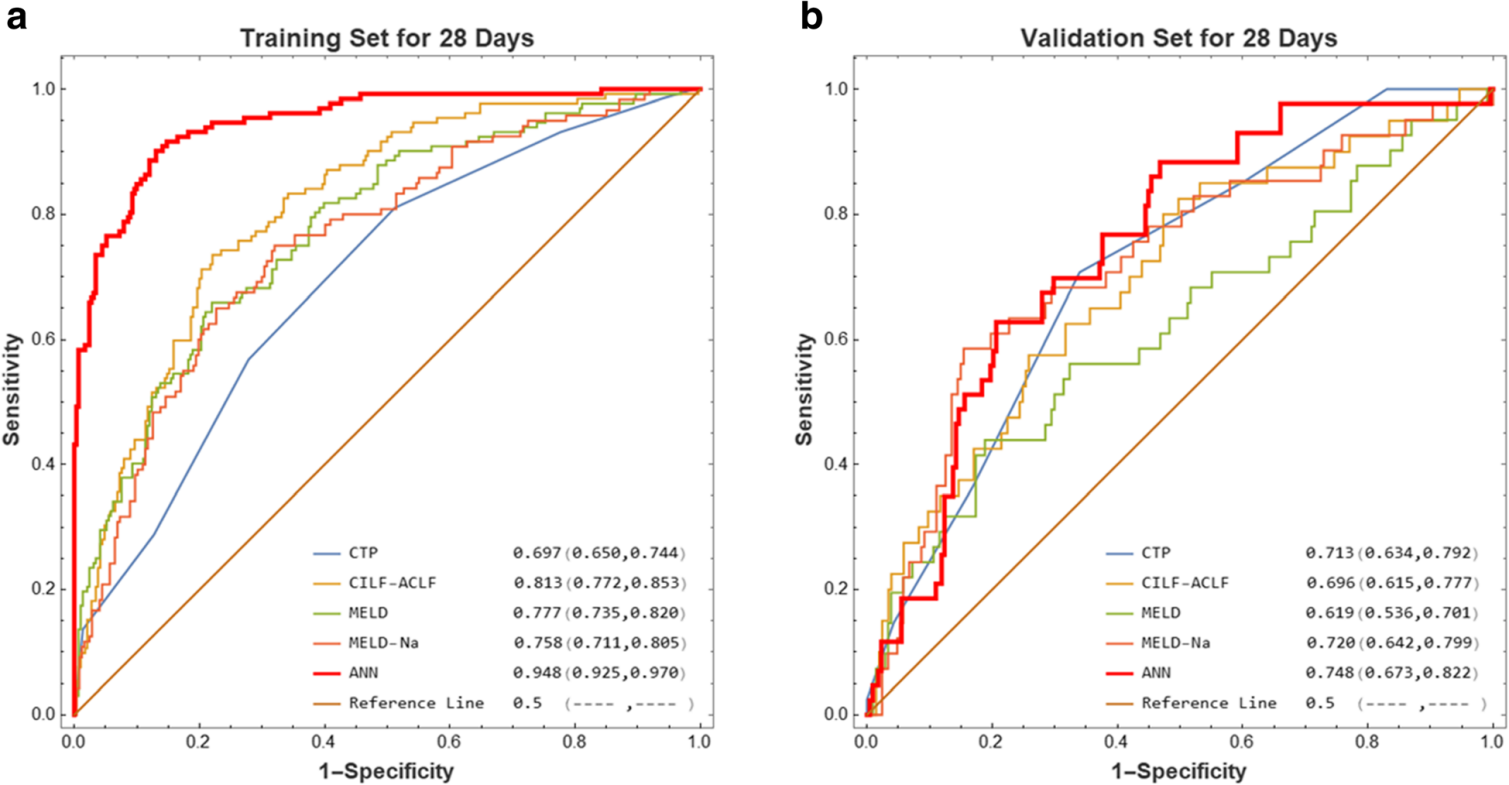
ROC analysis of MELD, MELD-Na, CTP, CILF-ACLF, and the constructed ANN model to predict 28-day mortality of HBV-ACLF in the (**a**) training and (**b**) validation cohorts, respectively

For 28-day mortality in the training cohort, the ANN model’s predictive accuracy (AUR 0.948, 95% CI 0.925–0.970) was significantly higher than that of MELD (AUR 0.777, 95% CI: 0.735–0.820, *p* < 0.001), MELD-Na (AUR 0.758, 95% CI: 0.711–0.805, *p* < 0.001), CTP (AUR 0.697, 95% CI: 0.650–0.744, *P* < 0.001), and CLIF-ACLF (AUR 0.813, 95% CI: 0.772–0.853, *p* < 0.001) (Fig. 3a). In the validation cohorts, the AUR of ANN model for 28-day mortality was 0.748 (95% CI: 0.673–0.822), which was still higher than that of MELD (AUR 0.619, 95% CI: 0.536–0.701, *p* = 0.0099), MELD-Na (AUR 0.720, 95% CI: 0.642–0.799, *p* = 0.424), CTP (AUR 0.713, 95% CI: 0.634–0.792, *p* = 0.303), and CLIF-ACLF (AUR 0.696, 95% CI: 0.615–0.777, *p* = 0.2004) (Fig. 3b).

**Fig. 4:**
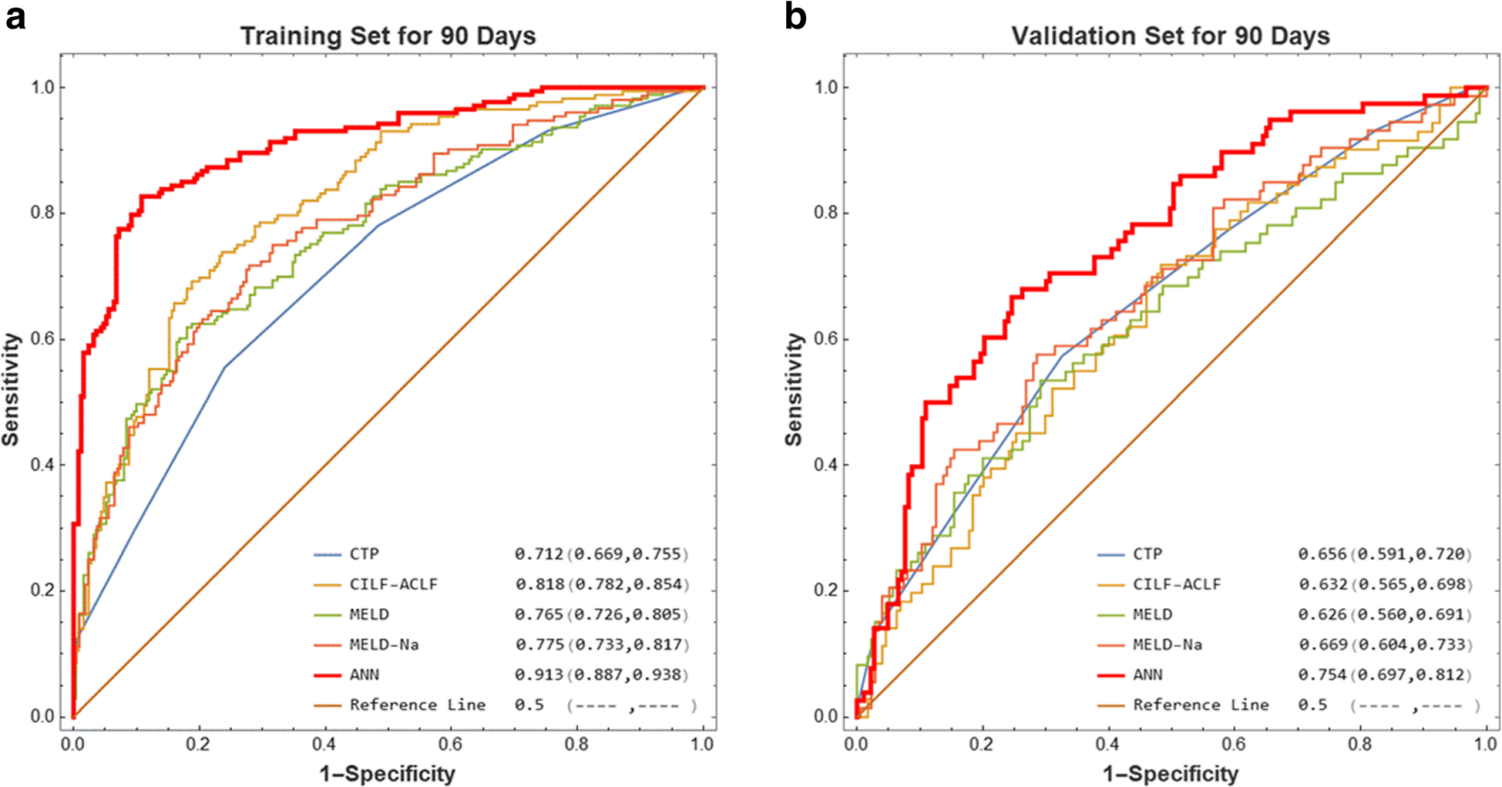
ROC analysis of MELD, MELD-Na, CTP, CILF-ACLF, and the constructed ANN model to predict 90-day mortality of HBV-ACLF in the (**a**) training and (**b**) validation cohorts, respectively

For 90-day mortality in the training cohort, the ANN model’s prediction accuracy (AUR 0.913, 95% CI 0.887–0.938) was significantly higher than that of MELD (AUR 0.765, 95% CI: 0.726–0.805, *p* < 0.001), MELD-Na (AUR 0.775, 95% CI: 0.733–0.817, *p* < 0.001), CTP (AUR 0.712, 95% CI: 0.669–0.755, *p* < 0.001), and CLIF-ACLF (AUR 0.818, 95% CI: 0.782–0.854, *p* < 0.001) (Fig. 4a). In the validation cohorts, the AUR of ANN model was 0.754 (95% CI: 0.697–0.812), which was still higher than that of MELD (AUR 0.626, 95% CI: 0.560–0.691, *p* = 0.019), MELD-Na (AUR 0.669, 95% CI: 0.604–0.733, *p* = 0.133), CTP (AUR 0.656, 95% CI: 0.591–0.720, *P* = 0.076), and CLIF-ACLF (AUR 0.632, 95% CI: 0.565–0.698, *p* = 0.264) (Fig. 4b).

## Discussion

MELD and other MELD-based scoring systems have been mainly using in patients with decompensated cirrhosis. However, since ACLF is different from liver cirrhosis in multiple aspects (e.g. mortality), MELD and other MELD-based scoring systems are not adequately enough to predict mortality of ACLF. In addition, compared with single factors, combination of multiple factors can be more effective in the prediction of prognosis of ACLF. Until now, there have some nomograms established to predict prognosis of ACLF. For example, Gao et al. combined neutrophil to lymphocyte ratio (NLR), HE, INR, age and TBIL to construct a prediction model for 90-day mortality of HBV-ACLF, which showed better predictive values in comparison with MELD, MELD-Na, and CTP [25]. Lei et al. proposed a formula model based on NLR, GGT, albumin, sodium and artificial liver support therapy to predict short-term mortality of HBV-ACLF [26]. Chen et al. combined age, serum sodium and MELD score to develop a prognostic nomogram to assess 3-month mortality of HBV-ACLF, which exhibited better efficacy than MELD, MELD-Na and iMELD [27]. Lin et al. developed a prognostic nomogram, on the basis of risk factors (including age, MELD and liver to abdominal area ratio), to predict 3-month mortality of HBV-ACLF, with higher prediction accuracy of 87.7% in comparison with MELD score, MELD-Na and CTP [28].

In contrast to multiple regression models combining variables significantly associated with mortality of HBV-ACLF, ANN is generally more suitable to model nonlinear complex biological systems. We first identified risk factors for mortality of HBV-ACLF. We showed significant differences in PTA, TBIL, age, serum sodium, ALP, HBeAg positivity rate, GGT and HE between the survivors and non-survivors both at 28 days and 90 days during the follow-up. It had been shown that PTA was associated with mortality of HBV-ACLF [29], and age, TBIL HE and serum sodium were also used to predict the prognosis of HBV-ACLF [7, 8, 25, 29, 30]. In addition, HBeAg negativity was shown to be significantly correlated with more severity of liver disease [31–33], showing a correlation of HBeAg positivity rate with better prognosis of liver disease. Our result was generally consist with those reported. Interestingly, we showed sodium and HBeAg positivity rate were negatively associated with 28-day mortality but were positively associated with 90-day mortality. There might be several reasons. First, the univariate analysis may be affected by confound factors because of interaction between variables. There should be difference in physiological status, physiological and biochemical parameters, etc. between the 28-day and 90-day mortality groups, which may cause difference in interference in the univariant analysis. As a result, parameters such as serum sodium and HBeAg positive rate may exhibit association with 28- and 90-day mortality in different way. Second, this might reflect the complexity of the prediction systems for the 28-day vs 90-day mortality.

We first carried out univariate analysis to preliminarily screen variables statistically associated with 28- and 90-day mortality of patients with HBV-ACLF. These variables with statistically significant difference or important clinical characteristics were input in the ANN training processes to select variables (a part of the variables in the training process) with contribution to significantly improve the predictive accuracy, which were eventually included in the ANN models. Those without significant contribution, such as white blood cell, INR and serum creatinine, were excluded. Therefore, only a part of the variables with statistically significant difference after univariate analysis were eventually included in the ANN models. The selection criteria of variables was to see if they could significantly increase the prediction efficacy of models. To produce more variables in the ANN training process and bring about more variables eventually including in the ANN models to get more satisfactory predictive efficacy, we performed single univariate analysis instead of extra multivariate analysis, and then directly included the variables with statistical difference into the ANN models. We thus established ANN-based models to predict the 28- and 90-day mortality of HBV-ACLF on a level of individual patients. The ANN models were trained and constructed in a large cohort of HBV-ACLF patients (*n* = 423) and were then validated in another independent cohort (*n* = 261). ROC analysis demonstrated that the ANN models had higher predictive accuracy for the 28- and 90- mortality in the training cohort compared with the MELD-based and other scoring systems, including MELD, MELD-Na, CTP and CLIF-ACLF. This may be attributable to the complicated, multidimensional and nonlinear advantages of ANN.

In this study, the prediction models were established and then cross-validated in different cohorts from different centers. This assures that the constructed models can be independently and effectively validated. With an inference process, the ANN can reduce errors caused by new data sets, as a result, the final form provided accurate prediction values of 28- and 90-day mortality risks in each patient, with higher score predicting higher risk of mortality of HBV-ACLF patients.

Until now, there has only one similar study reporting ANN-based model for predicting short-term mortality risk of HBV-ACLF [34]. In comparison with Zheng et al.’ study (402 cases in one hospital), the present study not only analyzed more (684) cases of patients but also involved more (eight) tertiary hospitals in different sites of China which may increase the statistical power and research reliability. Zheng et al. combined 6 variables including age, PTA, serum sodium, TBIL, HBeAg positive rate and hemoglobin to construct an ANN model for predicting 3-month mortality risk of HBV-ACLF, with a prediction accuracy of 86.9 and 75.5% in the training and validation cohorts, respectively [34]. In contrast, our study involved more subjects both in the training and validation cohorts, and included more (up to 8) variables (PTA, TBIL, age, NA, ALP, GGT, HE and HBeAg positive rate) to build ANN models used for predicting both 28- and 90-day mortality of HBV-ACLF, with a higher predictive accuracy of 91.3 and 81.8% for 90-day mortality in the training and validation cohorts, respectively. Establishment of ANN models are becoming popular. Generally, inclusion of more subjects, more variables, etc. may bring about more accuracy of models. In the present study we selected all the variables with statistical significance after the univariate analysis in the ANN training process to observe the predictive efficacy. We did find the eight variables, eventually included in the present ANN models, significantly increased the predictive efficacy of 28- and 90- day mortality of HBV-ACLF. The present result also supported that the more variables-based ANN models showed better predictive efficacy for mortality of HBV-ACLF than Zheng et al.’ study. In addition, we also built an ANN model used for predicting 28-day mortality risk, with a general satisfactory predictive accuracy of 94.8% in the training cohort and 74.8% in the validation cohorts, which has not been previously reported. Our ANN prediction models for mortality of HBV-ACLF can be available on line (https://zsg.github.io/ditan_zxyjh/).

The ANNs have obvious advantages in rapidly and accurately managing nonlinear complex biological systems. In the coming ‘Big Data’ era, large clinical data regarding the cases of HBV-ACLF patients can be shared from various medical centers, and enough related variables and large sample size will in turn make ANN models much more accurate to predict the prognosis of HBV-ACLF in the future.

There are some limitations in this study. First, the employed black-box solution (nonlinear mapping) in neural network models is a major drawback in determining possible internal correlations between input and output variables. Further analyses (such as sensitivity analysis) are thus needed to reveal their inference mechanism. Second, this was a retrospective study which might bring about bias in the case selection and incompletion of clinical information. Moreover, this was not a randomized study that patients in the training and validation cohorts had different mortality rates. The 28-day and 90-day mortality rates in the training cohort were significantly higher than in the validation cohort (28-day mortality 31.2% vs 16.5%, *P* < 0.001; 90-day mortality 40.9% vs 29.9%, *p* = 0.004, respectively). Generally, mortality rate in a cohort would be correlated with the prediction efficacy by models. It is thus not surprising that the prediction performance of the ANN in validation cohort is inferior to that in training cohort. Next, multicentered, prospectively designed, randomized studies involving more patients over longer follow-up period are needed to validate this result. Third, HBV infection-related factors (such as HBV-DNA level and HBeAg positive rate) were included in analysis, while some other HBV infection markers, such as HBsAg titers, were not evaluated in the present study since complete information for all patients was not available. In the future prospective study, more variables especially including more HBV markers (such as HBsAg titers) will be collected and analyzed. Fourth, multiple regression models combining several variables significantly associated with mortality of HBV-ACLF would improve the prediction efficacy of prognosis in comparison with single variables, while we did not try them. However, given the advantages of ANN models which are generally more suitable for analysis of non-linear data with much more complexity, we believe the established ANN models will be superior to other models (including multiple regression models). Next, multiple regression models and other models will be prepared to further validate the advantages of the ANN models in the prediction of 28- and 90-mortality of HBV-ACLF patients.

## Conclusions

In summary, we established ANN-based models for predicting 28- and 90-day mortality of HBV-ACLF, which exhibited superiority to the conventional MELD-based and other routine scoring systems. This study might be helpful to identify HBV-ACLF patients with poor prognosis so as to take treatment as early to achieve better outcomes.

## Data Availability

The dataset used and analysed during the study can be available from the corresponding author on reasonable request.

## Abbreviations

95% CI: 95% Confidence interval
ACLF: Acute-on-chronic liver failure
ALP: Alkaline phosphatase
ALT: Alanine aminotransferase
ANN: Artificial neural network
APASL: Asia Pacific Association for the Study of the Liver
AST: Aspartate aminotransferase
AUR: Area under the ROC curve
BP: Back propagation
CHB: Chronic HBV
CLIF-ACLF: Chronic Liver Failure-ACLF
CTP: Child-Turcotte-Pugh
GGT: Gamma-glutamyl transpeptidase
HBeAg: Hepatitis B e antigen
HBV: Hepatitis B virus
HBV-ACLF: HBV-associated ACLF
HE: Hepatic encephalopathy
INR: International normalized ratio
MELD: Model for End-stage Liver Disease
MELD-Na: MELD integrating sodium
MLP: Multilayer perceptron
NLR: Neutrophil to lymphocyte ratio
NLR: Neutrophil to lymphocyte ratio
NUCs: Nucleotide analogs
OR: Odds ratio
PTA: Prothrombin activity
ROC: Receiver operating characteristic curve
TBIL: Total bilirubin
